# Lightweight robust detection of anthropogenic floating debris in turbid and dynamic aquatic environments via enhanced feature fusion

**DOI:** 10.1038/s41598-025-31043-9

**Published:** 2025-12-03

**Authors:** Yuanzhuo Zhong, Jiaquan Wan, Mingzhu Cao, Zuowen Tan, Yanbin Qiu, Lvfei Zhang, Xinwu Ji, Leqi Shen, Tao Yang

**Affiliations:** 1 College of Hydrology and Water Resources, Hohai University, Nanjing, 210098 China; 2 College of Information, Shanghai Ocean University, Shanghai, 201306 P. R. China

**Keywords:** Anthropogenic floating debris, YOLO, Object detection, Complex interference, Ecology, Ecology, Engineering, Environmental sciences, Mathematics and computing

## Abstract

Accurate detection of Anthropogenic Floating Debris (AFD) is crucial for water pollution management. However, existing detection algorithms often lack environmental robustness in complex aquatic environments, and their high computational costs also impede deployment on lightweight platforms. To address these issues, this study proposes BiDB-YOLOv8, with an enhanced feature processing architecture. The model first employs a multi-branch convolutional block to improve the quality of extracted features, enhancing its ability to distinguish small targets from noisy backgrounds. It then uses an efficient bidirectional fusion network to ensure these high-quality features are effectively integrated across different scales. Additionally, this paper constructs and releases an original dataset, Turbid-floater, which includes diverse interference scenarios. Experimental results show that BiDB-YOLOv8 improves detection accuracy over the baseline YOLOv8n with a negligible increase in parameters. Compared to the larger YOLOv8s, it achieves nearly identical accuracy with only a fraction of the computational cost, highlighting a favorable trade-off between precision, robustness, and efficiency for real-world environmental monitoring.

**Supplementary Information:**

The online version contains supplementary material available at 10.1038/s41598-025-31043-9.

## Introduction

 Ensuring water security, alongside the restoration and protection of aquatic ecosystems, has been universally acknowledged as a core target within the United Nations Sustainable Development Goals (SDGs)^1^. However, widespread water pollution driven by accelerated industrialization and declining ecological resilience, has affected a large portion of global inland water bodies^2^. This pollution severely compromises the self-purifying capacity of aquatic ecosystems^3,4^ and resulting in irreversible ecological degradation^5^.

Among the diverse categories of aquatic pollution, *Anthropogenic Floating Debris (AFD)*^6^ poses increasing ecological and operational challenges^7,8^. Distinct from *Natural Floating Debris (NFD)*, AFD serves as a tangible indicator of urbanization-induced stress on water systems^9,10^, and is widespread on the surface of rivers, lakes, and reservoirs^11–13^. It leaches toxic substances^14^, absorbs heavy metals and organic toxins^15^, damages hydraulic infrastructure and disrupts aquatic ecosystems by blocking sunlight^16^. Furthermore, as many AFD items possess significant recycling value^17^, the rapid and accurate identification of such items is not only for mitigating pollution but also for recovering materials.

Timely detection and recognition of AFD is thus critical to the effectiveness of remediation efforts. Accurate automated monitoring significantly increases collection efficiency^18^ while minimizing ecological disturbance of manual retrieval^19^. However, current field implementation still relies on slow and labor-intensive manual methods^20^, ill-suited for real-time monitoring of large aquatic areas.

To address these constraints, computer vision and deep learning techniques have emerged as promising alternatives. Object detection, a foundational task in computer vision^21^, has shown great potential in environmental monitoring tasks such as floating debris detection^22,23^ and waste classification^24^. However, AFD detection presents unique challenges due to the confluence of multiple endogenous and exogenous interference factors. Targets are often small, visually ambiguous, and densely distributed (endogenous noise), while environmental variables such as fluctuating water surface conditions and light reflection constitute complex exogenous disturbances. These combined factors make robust detection extremely difficult in practice.

Among detection architectures, single-stage models like *YOLO (You Only Look Once)*^25^ series are ideal for the task. Their modular design and favorable trade-off between accuracy, speed, and architectural flexibility, making it an ideal foundation for the development of AFD-specific detection solutions.

Several studies have sought to improve YOLO-based detection systems for floating debris. Common strategies include adding multi-scale detection heads for small objects^26,27^, integrating attention mechanisms or enhanced fusion blocks to reduce background noise^12,28–30^, and applying novel data augmentation techniques to mitigate occlusion and reflection^31^. However, despite these advancements, three major limitations persist:

1. Many existing studies do not explicitly exclude NFD from detection, leading to reduced system efficiency and ecological risks, including unnecessary removal of beneficial organic debris^32,33^ and complications in pollution source tracing^34^.
2. Most models address either endogenous or exogenous interference in isolation, which limits robustness in real-world applications where both types frequently co-occur.
3. Several high-accuracy detectors overlook model compactness, restricting their deployment on embedded systems with constrained computational resources.

To address the deficiencies in current methodologies, recent works have proposed different design philosophies. Some adopt a modular assembly approach, combining separate components to target individual issues, as seen in MAE-YOLOv8^35^. Others, like APM-YOLOv7^36^, rely on external preprocessing to simplify the problem. In contrast, this study introduces BiDB-YOLOv8, an augmented detection model built upon the YOLOv8^37^ framework. The core of this model lies in a synergistic architectural design focused on the interplay between feature quality enhancement and efficient fusion. Achieving this synergy involves a two-step process: first, enriching feature maps with the Diverse Branch Block (DBB) directly addresses the visual ambiguity of small targets; second, ensuring these high-quality features are proficiently integrated by the Bidirectional Feature Pyramid Network (BiFPN) mitigates information loss across scales. This integrated approach facilitates accurate target detection amidst complex environmental disturbances. Moreover, this paper constructs and releases a customized AFD dataset that focuses on reflecting complex water surface conditions, ensuring the model can be trained and validated under conditions approaching real-world complexity, and providing data support for future related studies. The main contributions of this study are summarized as follows:

1. A Synergistically Optimized Network Architecture: This research presents a redesigned neck for the YOLOv8 model, creating a synergistic interplay between the feature extraction and fusion processes. To begin, by replacing standard convolutions in the C2f module with the structurally re-parameterized Diverse Branch Block (DBB), the model’s ability to capture contextual information and textural details across all scales is enhanced without increasing inference cost, leading to a marked improvement in the quality of feature inputs for the fusion network. Following this, the feature fusion network itself is radically transformed by adopting the Bidirectional Feature Pyramid Network (BiFPN) concept. Its bi-directional data flow and weighted fusion capabilities are employed to proficiently integrate the high-quality, multi-level features generated by the DBB. This combined strategy of “enhancing information quality” and “enabling efficient information fusion” ensures the model’s high performance when dealing with small objects and complex background interference.
2. An AFD Dataset for Complex Real-World Scenarios: This paper constructs and releases Turbid-floater, an original dataset containing diverse interference scenarios. It is characterized by the extensive inclusion of various endogenous and exogenous interference factors that may be encountered in real-world AFD detection tasks. This dataset aims to maximally enhance the model’s environmental robustness and generalization ability, as well as to provide reliable data support for similar research.
3. A Lightweight, Efficient, and Validated Detection Model: By integrating the above innovations, the proposed BiDB-YOLOv8 model outperforms several versions of the baseline YOLO and its variants across multiple key metrics, achieving an excellent balance between detection accuracy and computational cost. Comprehensive experimental validation confirms that the lightweight and high-performance BiDB-YOLOv8 is effective for AFD detection in challenging, high-interference aquatic settings, indicating its substantial potential for dependable real-world deployment.

## Methodology​​

### ​​Overview of YOLOv8​​

This study selects YOLOv8 as the baseline model, a state-of-the-art single-stage detector known for its notable architectural flexibility and strong balance between efficiency and precision. YOLOv8 offers five variants (N, S, M, L, X), differentiated by network depth and parameter size, but they all share the same core components: a backbone for extracting hierarchical features from input images, a neck for multi-scale feature fusion, and a detection head for final classification and bounding box regression. For this work, the lightweight YOLOv8n variant was chosen to optimize for deployment on resource-constrained platforms. However, its standard structure is not specifically optimized for AFD detection in diverse interference scenarios, leading to the improvements detailed in the following sections.

### Diverse branch Block​​

The diverse and complex water surface backgrounds pose significant challenges to the expressive capacity of convolutional kernels, requiring richer feature maps with more contextual information and fine-grained detail. However, the convolutions in the original YOLOv8 are fixed-scale, making the model struggle to capture such diverse features efficiently.

**Fig. 1:**
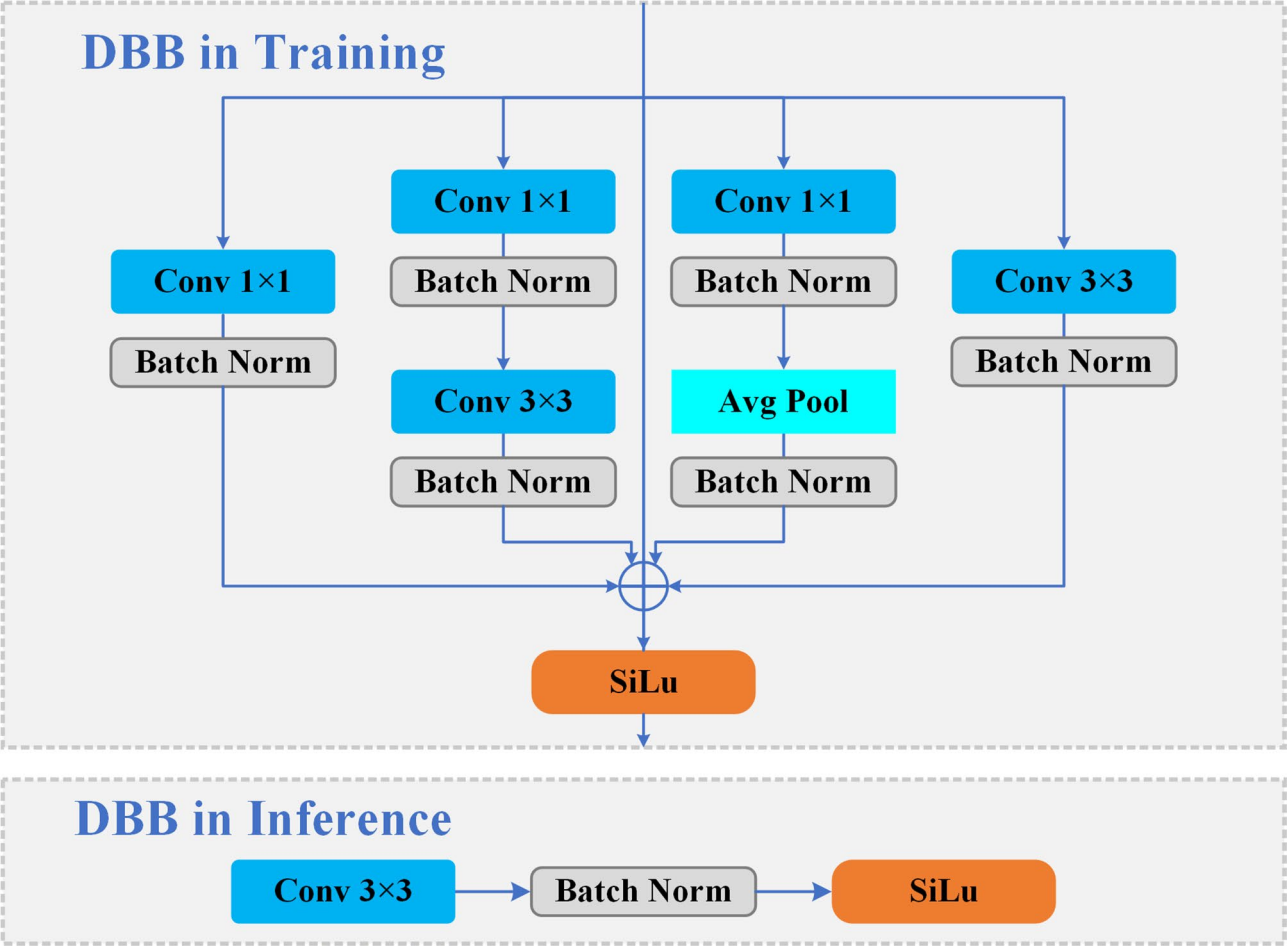
The architecture of the Diverse Branch Block (DBB). To enhance information quality from complex water surface backgrounds, DBB employs a multi-branch design during training (top panel), which enriches the feature maps. Critically, this complex training structure is equivalently converted into a simple and efficient single convolutional layer for inference (bottom panel). This strategy allows the model to leverage powerful multi-scale feature extraction during training while maintaining a lightweight and fast architecture during deployment.

To address this, this study introduces the Diverse Branch Block (DBB)^38^ as a replacement for the original convolutions, to enhance information quality. As illustrated in Fig. 1, DBB utilizes a multi-branch structure combining convolutions and pooling layers of various sizes and types, each branch focuses on capturing different spatial scales and feature types. This design, inspired by the Inception architecture^39^, offers the network a superior ability for contextual extraction and pattern capture under noisy conditions. Furthermore, DBB utilizes a structural re-parameterization mechanism. This allows the complex multi-branch training architecture to be equivalently converted into a single, standard convolutional layer during inference, which significantly mitigates the deceleration caused by larger convolutions.

Benefiting from its flexibility and modularity, DBB can theoretically replace any single convolution layer in the YOLOv8 architecture. To explore the optimal integration of DBB into the YOLOv8 architecture, its application was explored in three structural configurations: backbone_DBB, C2f_DBB, and Detect_DBB.

1. In backbone_DBB, all ConvModules in the backbone were replaced with DBB to enhance feature extraction by expanding the convolutional feature space.
2. In C2f_DBB, only the C2f modules in the neck were modified (illustrated in **Supplementary Figure ****S1**). Specifically, the two internal 3 × 3 ConvModules in each Bottleneck were replaced with DBB, aiming to improve multi-scale feature fusion.
3. In Detect_DBB, DBB was not only introduced to the detection head but also used to redesign its structure for better compatibility with the BiFPN neck, enabling more effective utilization of fine-grained features and improving small-object localization in complex environments.

A detailed variant optimization experiment is presented in **Sect. 4.2.1**. Based on the comparative analysis results, the C2f_DBB configuration was adopted for the final proposed model architecture.

### Bidirectional feature pyramid Network​​

**Fig. 2:**
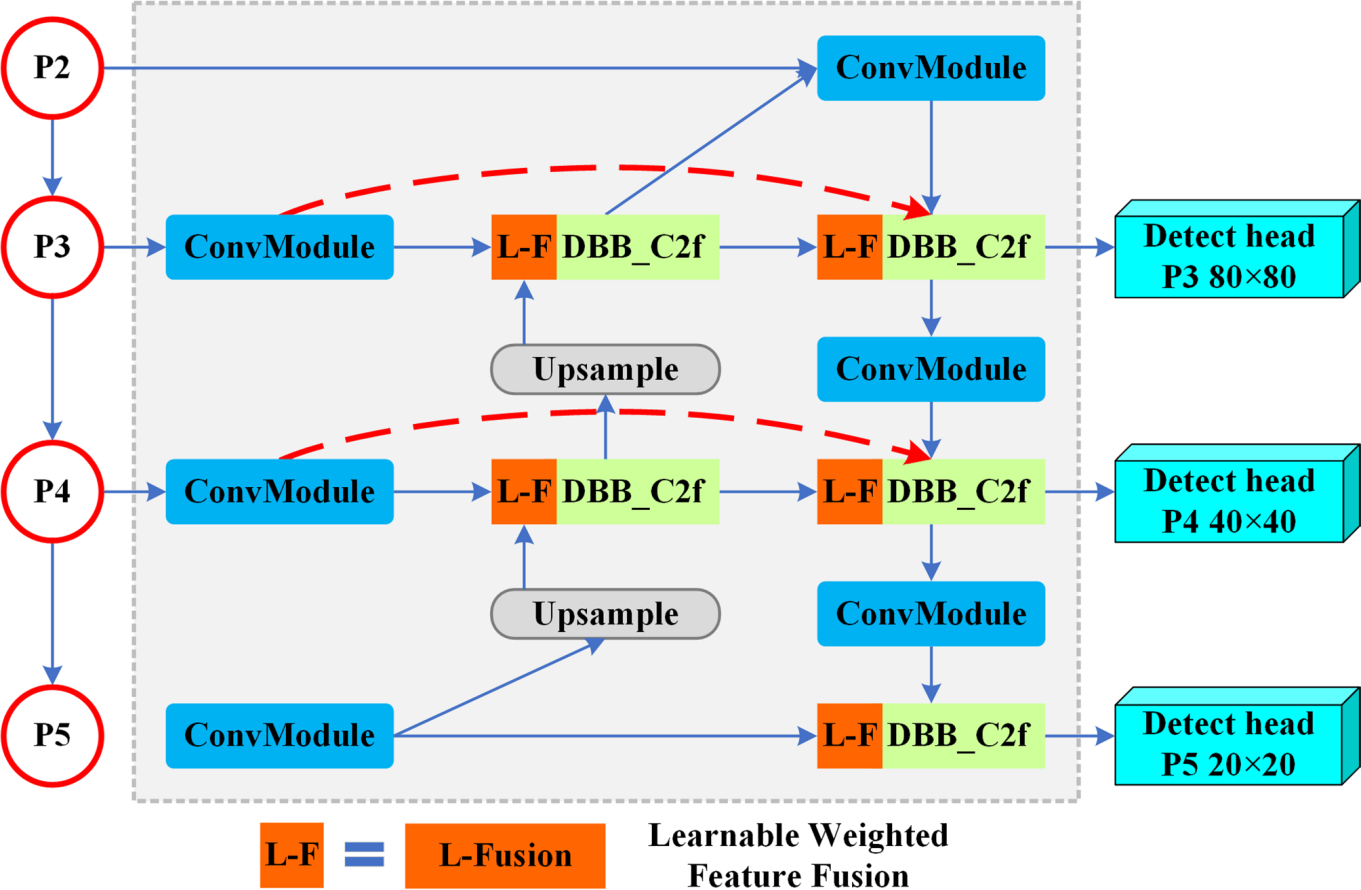
The architecture of the Bidirectional Feature Pyramid Network (BiFPN). To ensure that high-quality features from the DBB are fully utilized, the BiFPN architecture is adopted as a smarter fusion strategy. It enhances the model’s sensitivity to small and occluded objects by enabling a bidirectional information flow and preserving more details via cross-scale connections. Moreover, its weighted fusion mechanism (the L-F block) prioritizes more informative features, which is a core contribution to improving fusion efficiency while reducing parameter overhead.

After improving feature quality with DBB, it is essential to fuse these high-quality features effectively to ensure their full utilization. However, YOLOv8’s original feature fusion network, PANet, lacks specialized mechanisms tailored to small-object scenarios^35^, and thus may lose important details when combining features from different scales. To address this, this study redesigned the original neck by integrating a more efficient, adaptive and robust fusion architecture based on the Bidirectional Feature Pyramid Network (BiFPN)^40^, as a more effective feature fusion strategy shown in Fig. 2. BiFPN improves upon the original design in two main ways:

1. Efficient and lightweight structure: BiFPN enhances feature propagation by introducing lateral skip connections between nodes of equal resolution. This approach facilitates a richer and more coherent feature representation across scales, thereby ensuring the model’s sensitivity to small and occluded objects. Furthermore, BiFPN also simplifies the network by eliminating nodes that receive only a single input, as these contribute minimally to feature aggregation and increase computational redundancy.
2. Weighted feature fusion strategy: Instead of simply adding features together, a learnable weighted feature fusion strategy allows BiFPN to dynamically adjust the importance of each input feature, giving more weight to those with higher relative informativeness. This mechanism makes a core contribution to fusion efficiency, while reducing parameter overhead and computational cost.

By adopting BiFPN, the proposed model can make better use of the high-quality features generated by DBB, which effectively enhances both detection robustness and small-object sensitivity, leading to better localization and classification performance in challenging water environments.

### The improved Network​​

**Fig. 3:**
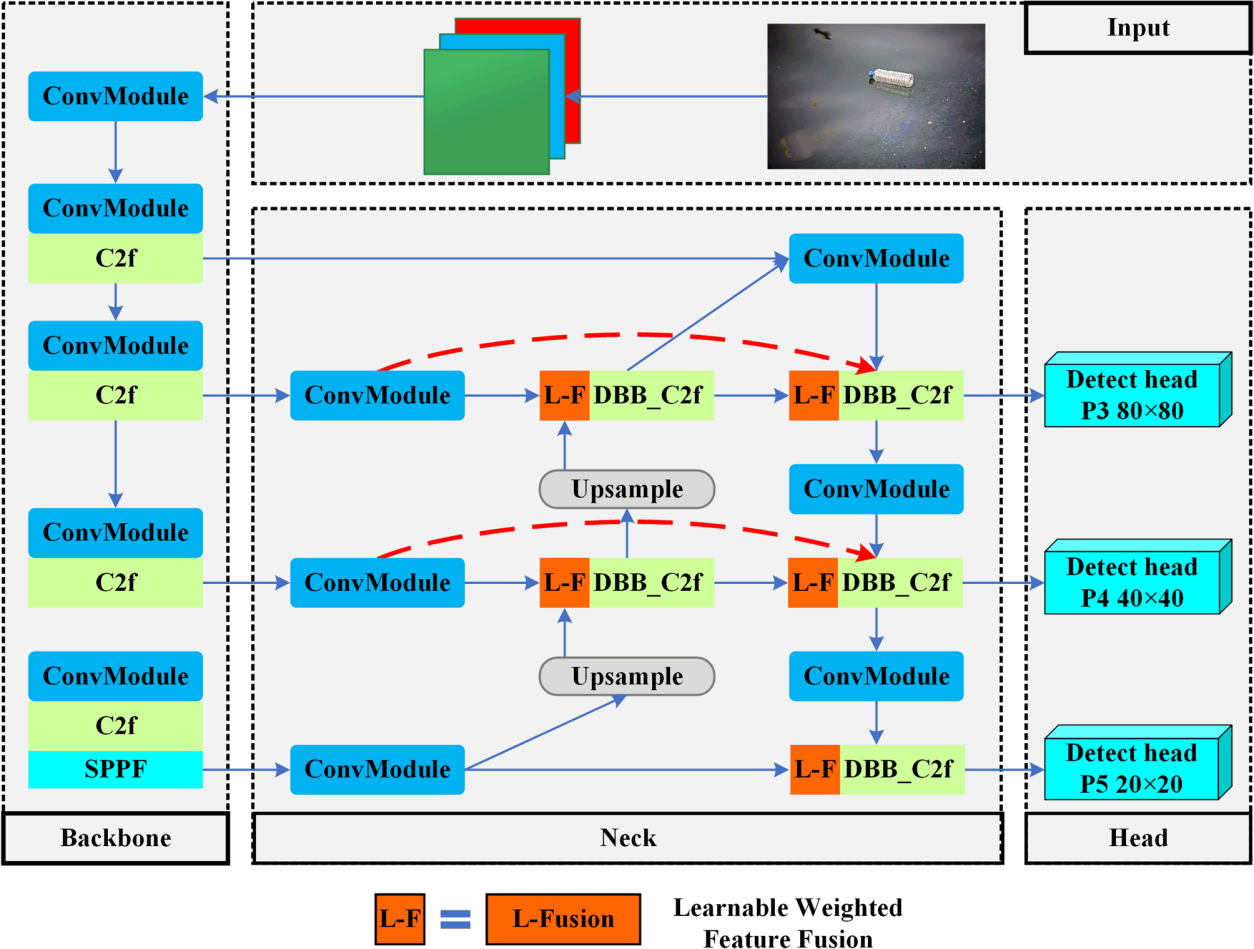
The synergistic architecture of BiDB-YOLOv8. The model’s core innovation is a tightly coupled workflow within its Neck. First, in the quality enhancement stage, features from the Backbone are refined by DBB-embedded C2f modules, enriching them for distinguishing small targets from background noise. Second, in the efficient fusion stage, the BiFPN structure intelligently aggregates these high-quality features, prioritizing the most salient information. This synergistic interplay ensures that enriched features are maximally preserved and utilized, leading to superior model robustness.

To overcome YOLOv8’s limitations in small-object detection and its sensitivity to complex environmental interference, this paper proposes BiDB-YOLOv8. As illustrated in Fig. 3, the architecture is built upon a synergistic design that directly embodies our strategy of “enhancing information quality” and “enabling efficient information fusion.” The operational flow is as follows:

1. Feature Extraction: The standard YOLOv8 backbone is in charge of initial feature extraction.
2. Quality Enhancement: As these features enter the neck, the DBB-embedded C2f modules further refine them using their multi-branch structure, enriching the feature maps for distinguishing small AFD from background noise.
3. Efficient Fusion: These refined, high-quality feature maps are then processed by the BiFPN. Its weighted fusion mechanism intelligently aggregates the multi-scale information, prioritizing the most salient features while minimizing information loss.

This tightly coupled design ensures that the high-quality information generated in the enhancement stage is maximally preserved and utilized during the fusion stage, leading to a model with superior robustness and accuracy for small-object detection in challenging environments.

## Experiment materials

### Dataset and dataset preprocessing

The dataset used in this study consists of two public datasets and a custom-built original dataset, *Turbid-floater*, which was specifically created to enhance sample diversity and simulate real-world conditions.

#### Public datasets

This study utilizes two public datasets as part of the training data, which suffer from issues such as data redundancy due to repetitive scene sampling and a lack of diversity in aquatic backgrounds. Full details of the public datasets used are provided in **Supplementary Section S1.**

#### Original custom AFD dataset: turbid-floater

To address the limitations of public datasets concerning sample diversity and background realism, an original dataset, *Turbid-floater*, was developed. This dataset was constructed by collecting images from Baidu, Google, Bilibili, and TikTok using keyword combinations such as “floating”, “debris”, “garbage”, and “waste”, retaining only publicly accessible results. Items were screened to keep visually identifiable recyclable man-made debris (e.g., plastic bottles, cans, glass bottles, cartons), and night or frozen-water scenes were excluded. All instances were annotated with YOLO.txt rectangular boxes (one file per image; one object per line in normalized class x_center y_center width height).

The web-sourced images referenced in this subsection are third-party works discovered via search engines and video platforms. The original images are not redistributed. Instead, we publicly release YOLO-format annotations and a provenance/URL manifest (platform and source-page identifiers; recovered source links where available) so that users can obtain images from the original hosts under the hosts’ terms/licences. All rights to the images remain with their respective rightsholders. The annotations and metadata only are released under CC BY 4.0, and a takedown contact is provided in the repository README. The annotations and the manifest are available at 10.5281/zenodo.15631053.

Designed to mirror complex real-world cleanup situations, the dataset features a range of challenges, such as low-resolution small targets (down to 14 × 14 pixels), occluded objects, NFD interference, turbid water, and diverse lighting and camera conditions. This high degree of heterogeneity is intended to enhance the model’s environmental adaptability. After data augmentation, the dataset was expanded to 1,454 images and divided into training and validation sets at a 7:1 ratio, and the visual differences among the datasets are shown in **Supplementary Figure ****S2****.** Furthermore, a quantitative summary of the Turbid-floater dataset is provided in **Supplementary Table ****S2**, detailing its composition and characteristics.

Finally, for a comprehensive and objective evaluation of the model’s robustness, a dedicated test set was constructed using images from all the previously mentioned data sources that were excluded from the training and validation stages.

### Evaluation indicators and experimental details

To comprehensively evaluate the effectiveness of both the model architecture enhancements and dataset optimization strategies, this study adopts the original YOLOv8 as the baseline and conducts a series of experiments based on the proposed BiDB-YOLOv8 model and the Turbid-floater dataset. The overall experimental arrangement, detailed definitions of all evaluation indicators, and a comprehensive list of implementation settings are provided in the **Supplementary Information (Supplementary Sections S2 and S3)**. In addition, we quantify uncertainty with 95% confidence intervals computed via image-level paired bootstrap (B = 1000, percentile interval). Further implementation specifics are provided in **Supplementary Section S3.**

## Experiment results

### Dataset effectiveness: impact of training data composition

Table 1 presents how different training data configurations affect the detection performance of YOLOv8n and YOLOv8s. Here, mAP@0.5_1_ and mAP@0.5_2_ correspond to results obtained when training solely on *IWHR-FloW* and *Turbid-floater*, respectively, while mAP@0.5_test_ indicates the performance on the test-set.

From Experiments 1 to 4, both model variants perform significantly better on *IWHR-FloW* (90.0 and 91.5) than on Turbid-floater (69.2 and 71.2). This contrast—when considered alongside the respective characteristics of each dataset—highlights a key limitation of the YOLOv8 series: poor detection of small objects under complex background interference. This issue serves as a primary motivation for the improvements proposed in this work.

Focusing on the mAP@0.5_test_ scores in these four experiments, only slight differences between models are observed, but a notable drop compared to mAP@0.5_1_ and mAP@0.5_2_. This suggests that training on a dataset with inherent bias fails to support learning of diverse feature patterns, thereby hindering the model’s generalization ability.

When both datasets are used jointly, as in Experiments 5 and 6, performance on the test-set improves considerably for both models. This indicates that combining diverse training samples effectively enhances generalization.

These results confirm that training with low-interference, single-domain datasets alone is insufficient for AFD detection in real-world scenarios. The Turbid-floater dataset addresses this gap by enriching feature diversity under noisy conditions, significantly improving model generalization and robustness, thereby offering practical support for related research tasks.

**Table 1:** Impact of training dataset composition on YOLOv8 model performance on the Test-Set.

No.	Model	IWHR-FloW	Turbid-floater	mAP@0.5_1_%	mAP@0.5_2_%	mAP@0.5_test_%
1	YOLOv8n	√		90.0		55.0
2	YOLOv8s	√		91.5		59.4
3	YOLOv8n		√		69.2	53.6
4	YOLOv8s		√		71.2	55.7
5	YOLOv8n	√	√			78.6
6	YOLOv8s	√	√			80.4

### Variant optimization

Integrating DBB and BiFPN into YOLOv8 introduces multiple structural variants. To reduce architectural uncertainty and establish clear baselines for ablation studies, a series of optimization experiments are conducted. These experiments systematically evaluated different structural configurations to identify the architecture with the best trade-off between performance and computational cost.

The experiment results show that, C2f_DBB is the most qualified DBB integration strategy, achieving the best trade-off between performance and parameter cost among all the variants. Furthermore, applying this modification to all five C2f modules within the BiFPN structure (termed the BiFPN + 5DBB variant) also outperformed other configurations on all evaluation indicators. Therefore, this optimized architecture was used for all subsequent ablation and comparative experiments. The detailed methodology, results, and analysis of these optimization experiments are provided in **Supplementary Section S4.**

### Ablation experiments

In water surface debris detection under complex backgrounds, it is essential to design improved models targeting small object characteristics and unpredictable interference. To evaluate the effectiveness of the proposed components—DBB and BiFPN—a set of ablation experiments was conducted. Besides those architectural components, the study also evaluated Shape-IoU as a potential optimization for the loss function. The results of all experiments are summarized in Table 2.

One of the first and most obvious conclusions is, the application of Shape-IoU is unsatisfactory. As detailed in Experiments 5 through 7 of Table 2, all model variants incorporating Shape-IoU showed a degradation in performance across both mAP@0.5 and mAP@0.5:0.95 metrics compared to their counterparts. This indicates that Shape-IoU does not provide a tangible benefit for AFD detection in complex aquatic environments. Consequently, it was excluded from the final improved model.

Introducing the BiFPN network enhances the transmission of feature information between input and output nodes at the same level through skip connections. The increased number of C2f modules further improves multi-scale feature fusion. As a result, the model achieves gains of 1.0% in mAP@0.5 and 1.2% in mAP@0.5:0.95 over the baseline. The notable improvement in the stricter mAP@0.5:0.95 indicates that the learnable weighted feature fusion mechanism successfully captures more suitable weight distributions for AFD detection under complex conditions, thereby improving generalization and robustness. At the same time, BiFPN reduces the parameter count by 0.23 M compared to the baseline, indicating that the newly designed fusion nodes and removal of redundant connections offset the computational cost introduced by additional C2f modules, achieving both efficiency and performance.

The DBB structure significantly enriches the convolutional space, enhancing the contextual learning capability of single convolution modules. When DBB is applied to the C2f modules in the neck, the model’s ability to learn multi-scale features improves substantially. As shown in Experiment 3, precision and mAP@0.5 increase by 2.5% and 0.8%, respectively, with an acceptable parameter overhead of 0.28 M.

Combining BiFPN and DBB yields the best overall performance. In Experiment 4, the model achieves improvements of 1.6% in mAP@0.5 and 2.0% in mAP@0.5:0.95, with only a 0.06 M increase in parameters. This demonstrates that the integration of DBB into C2f modules contributes directly to more effective feature fusion and enhances the model’s resistance to interference under high-confidence detection scenarios.

**Fig. 4:**
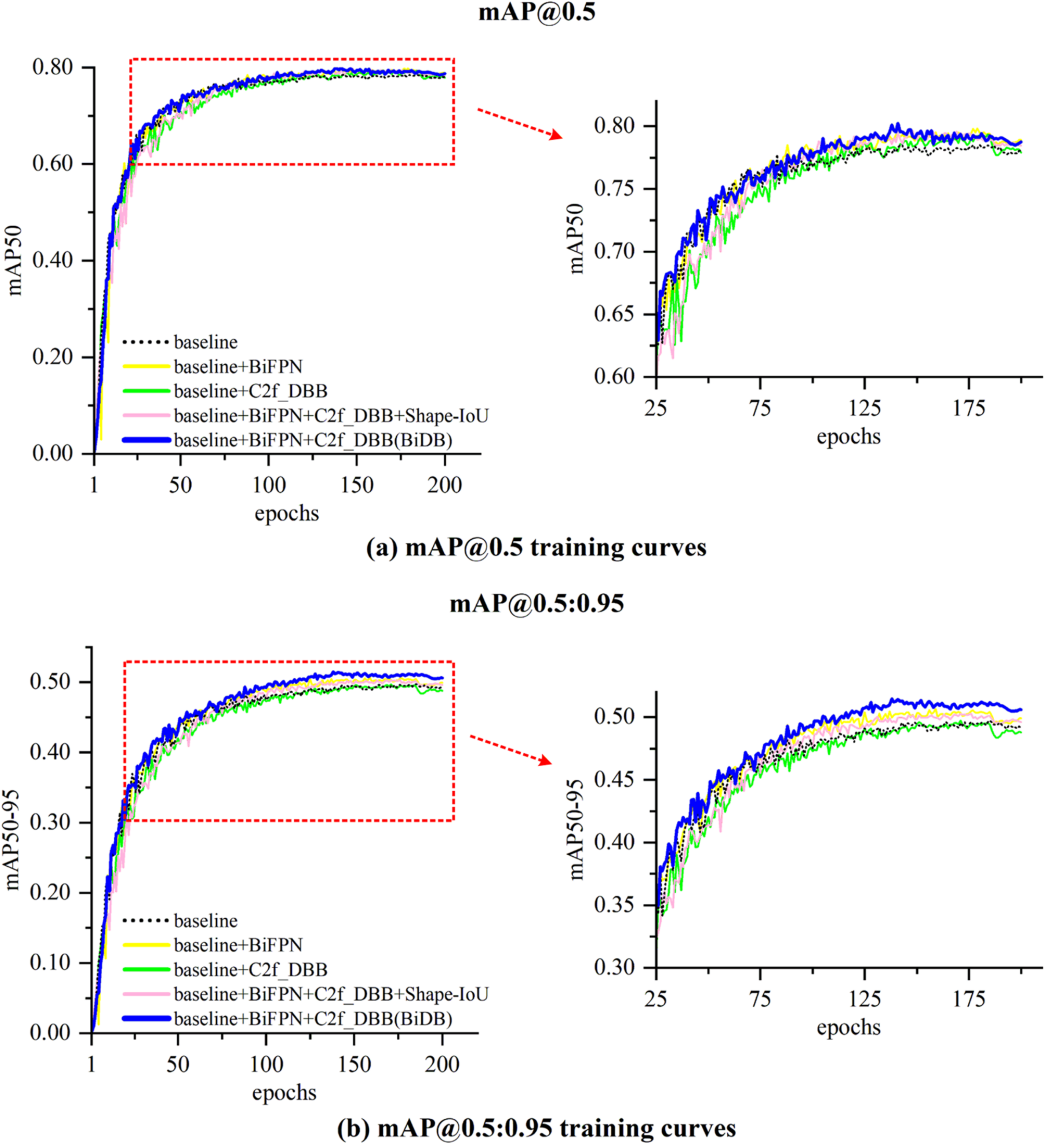
Training curves for ablation experiments in mAP@0.5 and mAP@0.5:0.95.

**Fig. 5:**
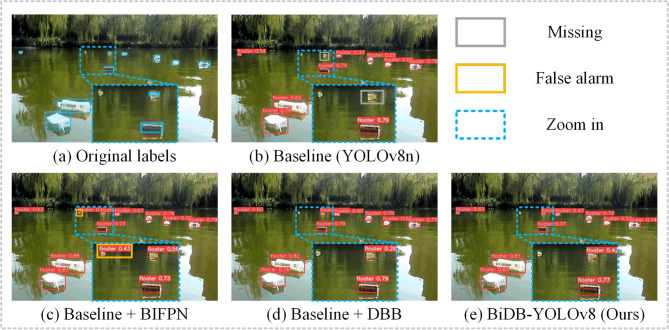
Ablation experiments Results.

Figure 4 illustrates mAP@0.5 and mAP@0.5:0.95 curves throughout the training process. BiDB-YOLOv8 maintains a consistent lead, particularly in mAP@0.5:0.95, further validating the effectiveness of the proposed improvements under stricter evaluation criteria. Figure 5 presents visual examples from the ablation experiments, including interference factors such as surface reflection, water mirroring, low resolution, and overexposure. All models incorporating either DBB or BiFPN successfully detect the yellow plastic bottles missed by the baseline. The combined model, BiDB-YOLOv8, achieves the highest detection confidence without false positives, effectively demonstrating its robustness and interference resistance.

The analysis additionally quantifies uncertainty using image-level paired bootstrap (B = 1000, percentile). YOLOv8n achieved mAP@50 = 0.784 with 95% CI [0.7505, 0.8170], while our model reached 0.805 with 95% CI [0.776, 0.836]. This indicates a modest improvement (+ 0.021 mAP points; +2.7% relative) with uncertainty appropriately reflected by CIs; we avoid judging significance from CI overlap alone.

In conclusion, the fused model integrating both BiFPN and DBB is adopted as the final improved architecture for AFD detection under complex conditions and is designated as BiDB-YOLOv8.

**Table 2:** Ablation experiments on test-set.

No.	baseline	BiFPN	C2f_DBB	Shape-Iou	*P*	*R*	mAP@_50_	mAP@_50 − 95_	Parameters
1	√				81.4	72.0	78.5	49.5	3.01 M
2	√	√			83.0	71.5	79.5	50.7	2.78 M
3	√		√		83.9	70.6	79.3	49.5	3.29 M
4	√	√	√		80.1	72.9	80.1	51.5	3.07 M
5	√	√		√	78.1	73.2	78.6	49.8	2.78 M
6	√		√	√	79.3	72.5	78.7	49.6	3.29 M
7	√	√	√	√	79.0	72.4	79.5	50.3	3.07 M

### Comparison to other methods

To further validate the performance of BiDB-YOLOv8, comparative experiments were conducted on the TEST-SET using seven end-to-end models: YOLOv8n, YOLOv8s, YOLOv5n, YOLOv5s, YOLOv9s, Swin-Transformer (Tiny) and EfficientDet-b0. We adopt YOLOv8n as the sole baseline due to its widespread use as a lightweight detector, while YOLOv8s/YOLOv5n/YOLOv5s/YOLOv9s, Swin-Transformer (Tiny), and EfficientDet-B0 are reference models chosen to cover prior- and current-generation YOLO, a compact Transformer backbone, and an EfficientDet family model at similar small-model scales.

As shown in Table 3, BiDB-YOLOv8 attains competitive accuracy—higher than YOLOv8n/YOLOv5n/YOLOv5s/Swin-Tiny/EfficientDet-B0, comparable to YOLOv8s, and lower than YOLOv9s; however, the accuracy of YOLOv9s is achieved at the largest computational cost among the reference models (39.6 GFLOPs in our setting), which limits its deployability relative to lightweight models.

Table 3 further reports two deployment-oriented quantities: the per-image computational cost (GFLOPs) and the weights memory computed from parameter counts under standard numeric precisions. For the lower-accuracy reference models, YOLOv8n and YOLOv5n sit in the very-low-compute regime but incur pronounced accuracy losses; YOLOv5s and Swin-Transformer (Tiny) require more parameters and computation yet still underperform BiDB-YOLOv8; EfficientDet-B0 shows the largest accuracy gap despite its compact design. In contrast, BiDB-YOLOv8 combines low computation (8.9 GFLOPs) and a small weights footprint (about 11.7 MB, 5.9 MB, and 2.9 MB under FP32, FP16, and INT8, respectively), forming a favorable compute–memory envelope that the above reference models do not match.

Compared with YOLOv8s, BiDB-YOLOv8 demonstrates a much better balance between accuracy and efficiency. It achieves nearly identical accuracy, with only a marginal decrease of 0.3 and 0.8% points in mAP@0.5 and mAP@0.5:0.95, respectively. In terms of efficiency, however, it dramatically reduces the parameter count from 11.14 M to 3.07 M and the computational cost from 28.6 to 8.9 GFLOPs. Compared with YOLOv9s, the accuracy gap is modest (2.3 and 2.5% points higher for YOLOv9s in mAP@0.5 and mAP@0.5:0.95, respectively), whereas BiDB-YOLOv8 requires roughly one-third of its parameters and about one-quarter of its computational cost. This trade-off is more aligned with real-world deployment needs where memory and compute budgets are limited.

**Table 3:** Comparison with other detection models on test-set.

No.	Model	mAP@_50_	mAP@_50 − 95_	Parameters	GFLOPs
1	BiDB-YOLOv8 (Our model)	80.1	51.5	3.07 M	8.9
2	YOLOv8n	78.5	49.5	3.01 M	8.7
3	YOLOv8s	80.4	52.3	11.14 M	28.6
4	YOLOv5n	72.3	42.8	1.90 M	7.7
5	YOLOv5s	76.3	46.5	7.20 M	24.0
6	YOLOv9s	82.4	54.0	9.70 M	39.6
7	Swin-Transformer(Tiny)	35.1	13.9	28.30 M	4.5
8	EfficientDet-b0	53.4	27.9	3.90 M	2.5

## Discussion

### Synergistic effect of architectural improvements

The real-world experimental results presented in **Sect. 4.3** demonstrate the outstanding environmental robustness of the proposed BiDB-YOLOv8 model in complex aquatic environments. This provides compelling evidence for the effective integration of the BiFPN and DBB within the model’s neck, the synergistic mechanism are as follows:

Firstly, by substituting standard convolutions with DBB in the C2f modules, the model is empowered to capture richer contextual information and finer textural details at each feature scale. This strategy significantly enhances the quality of the feature maps that are fed into the feature fusion network.

In addition, the redesigned feature fusion network, leveraging BiFPN, ensures that high-resolution details from shallow layers and rich semantic information from deep layers are fully and efficiently integrated through its bidirectional information flow paths and cross-scale connections, making maximum use of the enriched features generated by DBB. This architecture, combined with the learnable weighted feature fusion mechanism that actively suppresses background noise interference, ensures the model can effectively address the dual challenges of endogenous and exogenous interference.

The results of the ablation study quantitatively confirm this synergy: as the overall performance of BiDB-YOLOv8 surpasses the sum of improvements from any single module. To complement this with qualitative evidence, visualizations of feature activation heatmaps are provided in the Supplementary Information (**Supplementary Section S5**), which visually corroborate this synergistic effect. This combined evidence demonstrates that the strategy of enhancing information quality and enabling efficient information fusion represents a fundamental improvement for tackling the challenges of compound interference in real-world scenarios.

### Practical implications for environmental monitoring

The proposed model’s balance of accuracy and efficiency directly addresses key operational bottlenecks in real-world AFD monitoring. Firstly, its enhanced robustness ensures reliable detection in challenging conditions like glare and turbidity. The analysis confirms the model’s performance patterns under such interference, allowing for the design of optimized deployment strategies, such as avoiding low-visibility periods (see **Supplementary Table ****S1**). This reliability is critical for effective cleanup operations and long-term pollution tracking.

Secondly, the model’s lightweight design (8.9 GFLOPs; ~5.9 MB at FP16), as detailed in **Sect. 4.4**, makes it highly suitable for resource-constrained platforms like UAVs and USVs. This computational efficiency extends mission duration and lowers the hardware cost, making automated monitoring technology more accessible to environmental agencies and researchers. Ultimately, this work offers a practical tool that translates algorithmic improvements into scalable solutions for environmental management.

### Limitations and future outlook

While the present study shows promising results, further refinements can be made to the data labeling strategy and the experimental design.

To prioritize robustness, considering the environmental complexity of the model’s target application, this study strategically consolidated all high-recyclability AFD into a single ‘floater’ class. This decision, however, inevitably sacrificed the model’s finer discrimination capability. Therefore, the foremost objective for future work is to develop and label a multi-class detection dataset. This will be critical to overcoming the single-label limitation that currently hinders the model’s utility in more specialized applications.

Another limitation stems from how the dataset was built. Although collecting images from multiple sources for the *Turbid-floater* dataset increased its diversity, the variations (heterogeneity) in cameras, image compression, and viewing angles could lead to a potential distribution bias.

Furthermore, a limitation of this study is the absence of direct on-device performance metrics. This study acknowledge the importance of computational performance indicators like FPS and latency for evaluating real-world deployability. The acquisition of these metrics, however, necessitates access to the target embedded platforms, such as UAVs or USVs, which were not available during the course of this research. Consequently, our analysis was constrained to a laboratory environment. Validating these theoretical efficiencies through direct, on-platform benchmarking is a designated priority for the next phase of this research.

## Conclusion

This study proposes an efficient and lightweight AFD detection algorithm, named BiDB-YOLOv8, designed for application under conditions of complex water surface interference and limited computational resources to address prominent challenges in current riverine pollutant management. First, by integrating the DBB structure into the C2f module, the model’s feature extraction capability was successfully enhanced at a minimal computational cost. Second, the neck structure was redesigned based on the BiFPN concept, strengthening the model’s capacity for cross-scale information flow and fusion. Furthermore, this work presents a publicly available, original dataset that reflects complex aquatic conditions, aimed at improving model generalization and providing data support for similar research.

Experimental results show that our model exhibits a highly competitive trade-off between detection accuracy and computational expense when compared with other mainstream object detection algorithms. The model not only outperforms YOLOv8n, YOLOv5n, YOLOv5s, and Swin-Tiny across both mAP@0.5 and mAP@0.5:0.95 metrics, but also achieves performance comparable to the YOLOv8s model with roughly a quarter of the computational cost and parameters. Application results in real-world scenarios further validate that BiDB-YOLOv8 can accurately capture small AFD targets amidst complex background interference, exhibiting satisfactory robustness and applicability for deployment on lightweight, autonomous surface inspection platforms. A limitation of this study is that, to ensure robustness, all recyclable AFD were consolidated into a single ‘floater’ class, which restricts the model’s ability to differentiate between material types. Future work will focus on expanding the dataset to support multi-class detection for targeted recycling and validating the model’s long-term stability on real-world inspection platforms.

## Supplementary Information

Below is the link to the electronic supplementary material.

Supplementary Material 1

## Data Availability

Public datasets used here are accessible at [*IWHR_AI_Label_Floater_V1* [http://123.56.14.89:8008/wfdownload/](http:/123.56.14.89:8008/wfdownload)] and [*FloW-Img* https://github.com/ORCA-Uboat/FloW-Dataset] (§3.1.1). For Turbid-floater (§3.1.2), annotations and a provenance manifest are released at [https://doi.org/10.5281/zenodo.15631053] ([CC BY 4.0/CC0]); original web-sourced images are not redistributed and should be obtained from the source hosts under their terms/licences. Peer-review access to image evidence is available on request.
