# Supplementary material for: Lightweight robust detection of anthropogenic floating debris in turbid and dynamic aquatic environments via enhanced feature fusion
Source: Sci Rep. 2025 Dec 3;16:1373. doi: 10.1038/s41598-025-31043-9 (PMC12796399; doi:10.1038/s41598-025-31043-9)
Supplement: Supplementary file 1 — Supplementary Material 1 [file 41598_2025_31043_MOESM1_ESM.docx]

Supplementary Information for:

# BiDB-YOLOv8: Robust Detection of Anthropogenic Floating Debris in Turbid and Dynamic Aquatic Environments via Enhanced Feature Fusion

Yuanzhuo Zhong, Jiaquan Wan*, Mingzhu Cao, et al.

## Supplementary Figure S1. The Structure of the C2f_DBB

The two internal 3×3 ConvModules in each Bottleneck were replaced with DBB, aiming to improve multi-scale feature fusion.


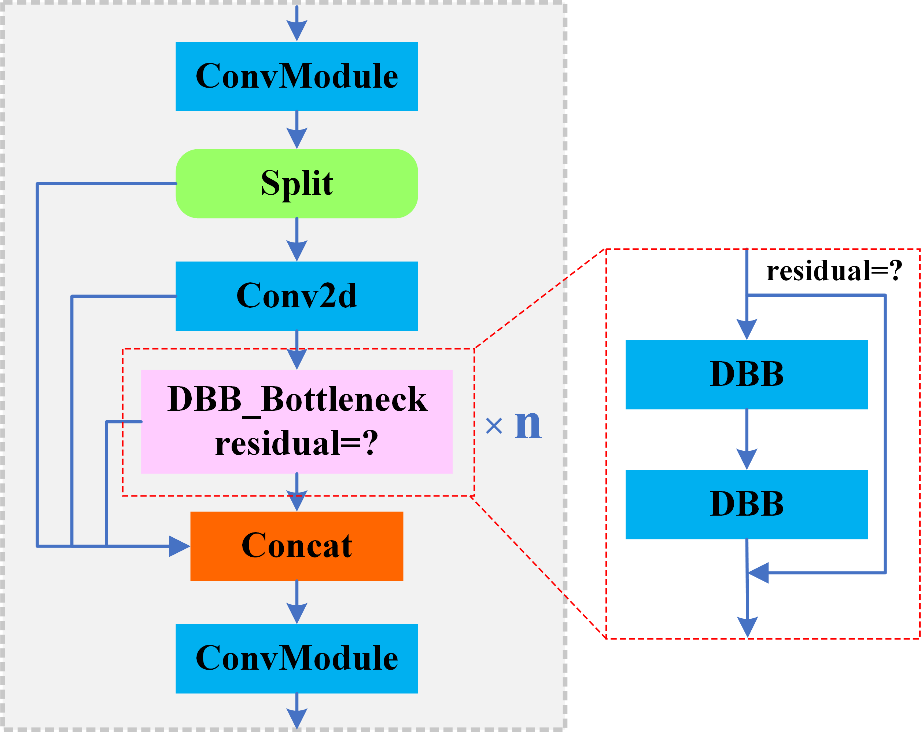


## Supplementary Figure S2. Image comparison from all three datasets


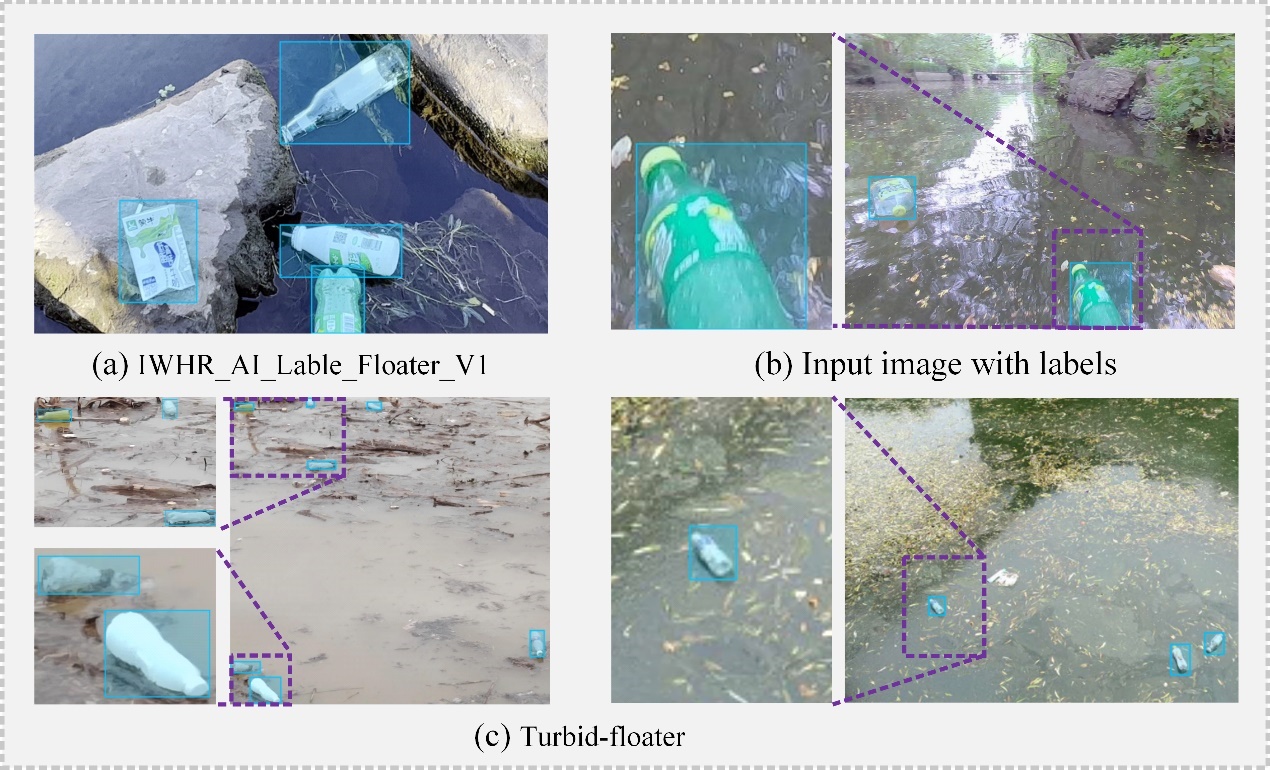


## Supplementary Table S1. Image comparison from all three datasets

**Supplementary Table S1**

Condition-stratified performance on the TEST-SET (BiDB-YOLOv8).

| No. | Test-set type | subset share | P | R | mAP_50_ | mAP_50-95_ |
| --- | --- | --- | --- | --- | --- | --- |
| 1 | Original | 100% | 80.1 | 72.9 | 80.1 | 51.5 |
| 2 | Blur | 27.5% | 79.6 | 65.1 | 74.5 | 46.4 |
| 3 | Over-exposure | 21.1% | 84.4 | 77.9 | 82.9 | 52.6 |
| 4 | Turbidity | 50.7% | 79.0 | 69.3 | 76.1 | 48.2 |
| 5 | specular glare | 41.5% | 81.8 | 77.0 | 81.9 | 52.4 |

## Supplementary Table S2. Quantitative summary of the Turbid-floater dataset

**Supplementary Table S2**

Statistical Overview of the Turbid-floater Dataset

| Statistic Category | Metric | Value / Description |
| --- | --- | --- |
| Basic Counts | Total Images | 1,454 |
|  | Dataset Split | Training: 1,272 images  Validation: 182 images |
|  | Total Instances | 13923 |
| Image Properties | Image Resolution  Range | Min: [156 × 110],  Max: [e.g., 3840 × 2160]  (W×H, in pixels) |
| Content Character | Challenging Conditions | Features a wide range of real-world challenges. A quantitative breakdown of these conditions on the representative test set is provided in Supplementary Table S1. |
|  | Target Size | Includes challenging small targets (down to 14×14 pixels), with a primary focus on small-to-medium object scales. |
|  | Image Source | Collected from diverse online platforms including Baidu, Google, Bilibili, and TikTok. |

## Supplementary Section S1. Public Datasets

This study utilizes two public datasets: *IWHR_AI_Label_Floater_V1(Yang et al., 2023)* and *FloW-Img(Cheng et al., 2021)*, which feature high-clarity images with minimal background interference. However, these datasets suffer from issues such as data redundancy due to repetitive scene sampling and a lack of diversity in aquatic backgrounds, limiting its generalization capability in real-world scenarios.

The two public datasets are combined in subsequent experiments, called *IWHR-FloW*, which contains 2,444 training images and 518 validation images and characterized by medium-to-high overall quality and relatively low environmental noise.

While this combined dataset provides a solid baseline, its relatively clean and uniform scenarios are insufficient to train a model robust enough for the chaotic conditions of real-world turbid waters.

## Supplementary Section S2. Evaluation Indicators

This study adopts Precision (P), Recall (R), and Mean Average Precision (mAP) as the primary evaluation metrics for object detection. The calculation formulas are as follows:

$$\begin{aligned} P=\frac{TP}{TP+FP}\#\left( 7 \right) \end{aligned}$$

$$\begin{aligned} R=\frac{TP}{TP+FN}\#\left( 8 \right) \end{aligned}$$

$$\begin{aligned} AP=\int_{0}^{1} P\left( R \right) dr\#\left( 9 \right) \end{aligned}$$

$$\begin{aligned} mAP=\frac{1}{N}\sum_{i=1}^{N} AP_{i}\#\left( 10 \right) \end{aligned}$$

​​ Where P represents the proportion of true positive predictions among all positive predictions, and R indicates the proportion of true positives among all actual positive cases. Specifically, TP, FP, and FN refer to true positives, false positives, and false negatives, respectively.

The mAP metric is defined as the weighted mean of Average Precision (AP) across all categories. This study uses both mAP@0.5 and mAP@0.5:0.95 as the overall evaluation indicators, with a weighted average ratio of 0.3:0.7 to select the optimal training epoch. Here, mAP@0.5 evaluates performance using a fixed IoU threshold of 0.5, while mAP@0.5:0.95 averages AP scores over IoU thresholds ranging from 0.5 to 0.95.

As all high-recyclability floating debris commonly found in river environments are treated as a single detection category during training, mAP is equivalent to AP in this context. AP is obtained by integrating the area under the Precision–Recall (P–R) curve. A higher AP value indicates better overall detection performance, reflecting a more favorable trade-off between precision and recall.

In addition to accuracy-based metrics, this study also incorporates the number of model parameters (Parameters) as an evaluation criterion to assess the model’s suitability for lightweight deployment on compact water surface monitoring devices.

## Supplementary Section S3. Experimental Details

To comprehensively evaluate the effectiveness of both the model architecture enhancements and dataset optimization strategies, this study adopts the original YOLOv8 as the baseline and conducts a series of experiments based on the proposed BiDB-YOLOv8 model and the Turbid-floater dataset.

In Section 4.1, the dataset effectiveness is evaluated by training the baseline and YOLOv8s models on different training set combinations to assess the impact of training data variations.

Section 4.2 explores integration strategies through variant optimization experiments, aiming to eliminate uncertainties prior to ablation.

Section 4.3 presents ablation experiments to quantify the contribution of each individual modification and determine the optimal model structure. In addition to point estimates, Section 4.3 reports 95% confidence intervals computed via an image-level, paired bootstrap (B = 1000, percentile), where both the baseline and the proposed model are evaluated on the same resampled image sets to control for item difficulty.

Finally, Section 4.4 introduces comparative models to further demonstrate the superiority of BiDB-YOLOv8.

All experiments are conducted on a laptop equipped with a 13th Gen Intel Core i5-13400 processor, NVIDIA GeForce RTX 4050 Ti (6 GB), 32 GB RAM, and Windows 11. The software environment includes Python 3.9.2, PyTorch 1.12.1, and CUDA 11.6. To ensure result consistency and reliability, uniform hyperparameters listed in **Supplementary Table S3** are applied across all experiments. All models, including all proposed variants, were trained end-to-end, meaning all modules such as DBB and BiFPN were optimized jointly from scratch.

**Supplementary Table S3**

Hyperparameter configurations

| No. | Parameters | Value |
| --- | --- | --- |
| 1 | Epochs | 200 |
| 2 | Batch | 16 |
| 3 | Image size | 640 |
| 4 | Workers | 4 |
| 5 | Cache | disk |
| 6 | Close mosaic | 15 |
| 7 | Optimizer | SGD |
| 8 | Momentum | 0.9 |

## Supplementary Section S4 Variant Optimization

### Supplementary Section S4.1 DBB Structural Optimization

Since all ConvModules in YOLOv8 can potentially be replaced with DBB, the impact of DBB placement is assessed by evaluating three model variants: backbone_DBB, C2f_DBB(Illustrated in **Supplementary Figure S1**), and Detect_DBB. All models are trained using the mixed *IWHR-FloW* + *Turbid-floater* dataset and compared to the baseline YOLOv8n.

As shown in **Supplementary Table S4**, backbone_DBB yields only a slight recall improvement (+0.3%) but shows a drop in both precision (–1.7%) and mAP@0.5 (–0.3%), with a parameter increase of 0.51M. This suggests limited benefit from applying DBB in the backbone.

In contrast, C2f_DBB and Detect_DBB deliver notable performance gains. C2f_DBB improves mAP@0.5 by 0.7% and precision by 5.7%, while Detect_DBB achieves respective gains of 0.2% and 2.3%. These results confirm the effectiveness of DBB in the neck and head for small-object detection under complex backgrounds.

Among the three, C2f_DBB achieves the best trade-off between performance and parameter cost. Since the neck is responsible for multi-scale feature fusion, expanding its feature space through DBB proves effective. As seen in **Supplementary Figure S3(a),** C2f_DBB maintains stable mAP@0.5 after 150 epochs, avoiding the performance fluctuations observed in other variants. **Supplementary Figure S3(b)** further shows its superior precision-recall performance in high-recall regions, which is essential for reducing false negatives in environmentally sensitive water bodies.

Based on these findings, C2f_DBB is selected as the preferred DBB integration strategy.


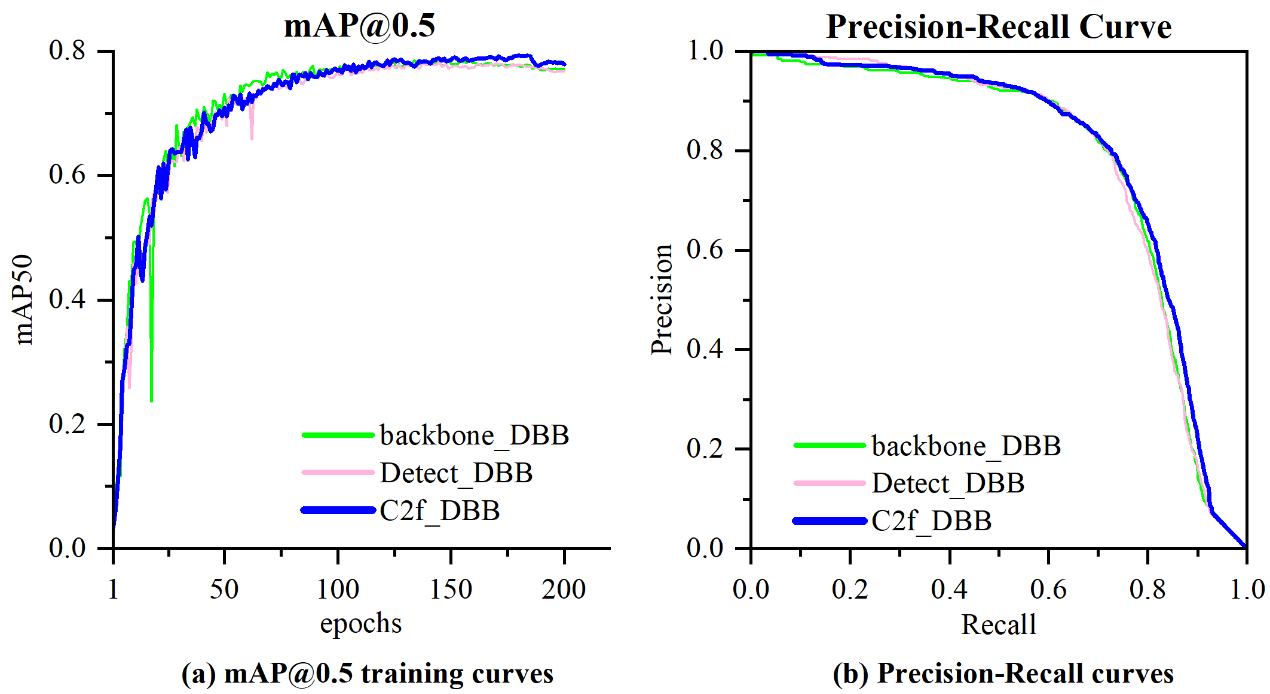


**Supplementary Figure S3.** **Precision–Recall curves and mAP50 training curves for different DBB variants**

**Supplementary Table S4**

COMPARISON OF ORIGINAL YOLOV8 AND 3 DBB VARIANTS

| No. | baseline | backbone_DBB | C2f_DBB | Detect_DBB | P | R | mAP_50_ | Parameters |
| --- | --- | --- | --- | --- | --- | --- | --- | --- |
| 1 | √ |  |  |  | 81.4 | 72.0 | 78.5 | 3.01M |
| 2 | √ | √ |  |  | 79.7 | 72.3 | 78.2 | 3.52M |
| 3 | √ |  | √ |  | 83.9 | 70.6 | 79.3 | 3.29M |
| 4 | √ |  |  | √ | 80.5 | 71.2 | 78.7 | 5.69M |

### Supplementary Section S4.2 BiFPN Network Integration

Both the BiFPN and C2f_DBB improvements target the neck of the YOLOv8 architecture. Since their areas of influence overlap, multiple integration strategies are possible. To ensure optimal performance when embedding C2f_DBB within the BiFPN-enhanced neck, designing comparative experiments in this section to identify the most effective fusion scheme.

The original YOLOv8 employs PANet in the neck for multi-scale feature fusion. When replaced with BiFPN, the number and position of C2f modules are altered, introducing uncertainty for DBB integration. Specifically, BiFPN adds an additional C2f module to the upsampling path to improve feature integration between the P3 and P4 layers, while the downsampling path remains unchanged. Based on whether C2f_DBB is inserted into the upsampling path, two BiFPN-based variants are constructed, as illustrated in **Supplementary Figure S4.** In the first variant, BiFPN+3DBB, C2f_DBB is applied only to the downsampling path. In the second, BiFPN+5DBB, C2f_DBB is applied to both upsampling and downsampling paths.

**Supplementary Table S5** presents the evaluation results of both variants on the test-set after training on the combined *IWHR-FloW* and *Turbid-floater* datasets. For reference, models using only one of the two improvements are also included.

Comparing Experiments 1, 2, and 4, the BiFPN+5DBB variant achieves a recall of 72.9% and an mAP@0.5 of 80.1%, outperforming both the standalone C2f_DBB and BiFPN models. This demonstrates the success of combining the two strategies, yielding improved performance in AFD detection under complex interference conditions. From the perspective of water safety and environmental protection, prioritizing higher recall facilitates the identification of more recyclable debris, which is more beneficial for real-world cleanup efforts. Hence, a moderate trade-off in precision is considered acceptable. In terms of computational cost, BiFPN+5DBB reduces the parameter count by 7% compared to only C2f_DBB, effectively combining DBB’s enhancement of convolutional expressiveness with BiFPN’s structural optimization for feature fusion.

Further comparison between the two variants shows that Experiment 4 exceeds Experiment 3 by 1.2% in recall and 0.9% in mAP@0.5, indicating that insufficient fusion in the upsampling path can directly degrade detection accuracy, while structural changes in this path do not negatively impact C2f_DBB integration. Additionally, differences between Experiments 2 and 3 reveal that partial embedding of C2f_DBB can even reduce performance. This suggests that maintaining structural consistency between upsampling and downsampling paths is essential. It is likely that the inconsistency disrupts the learnable weighted feature fusion mechanism, preventing the model from learning optimal weights for different feature levels, thereby affecting overall detection accuracy.

In conclusion, the 5DBB variant is adopted as the unified fusion strategy for integrating BiFPN and DBB.


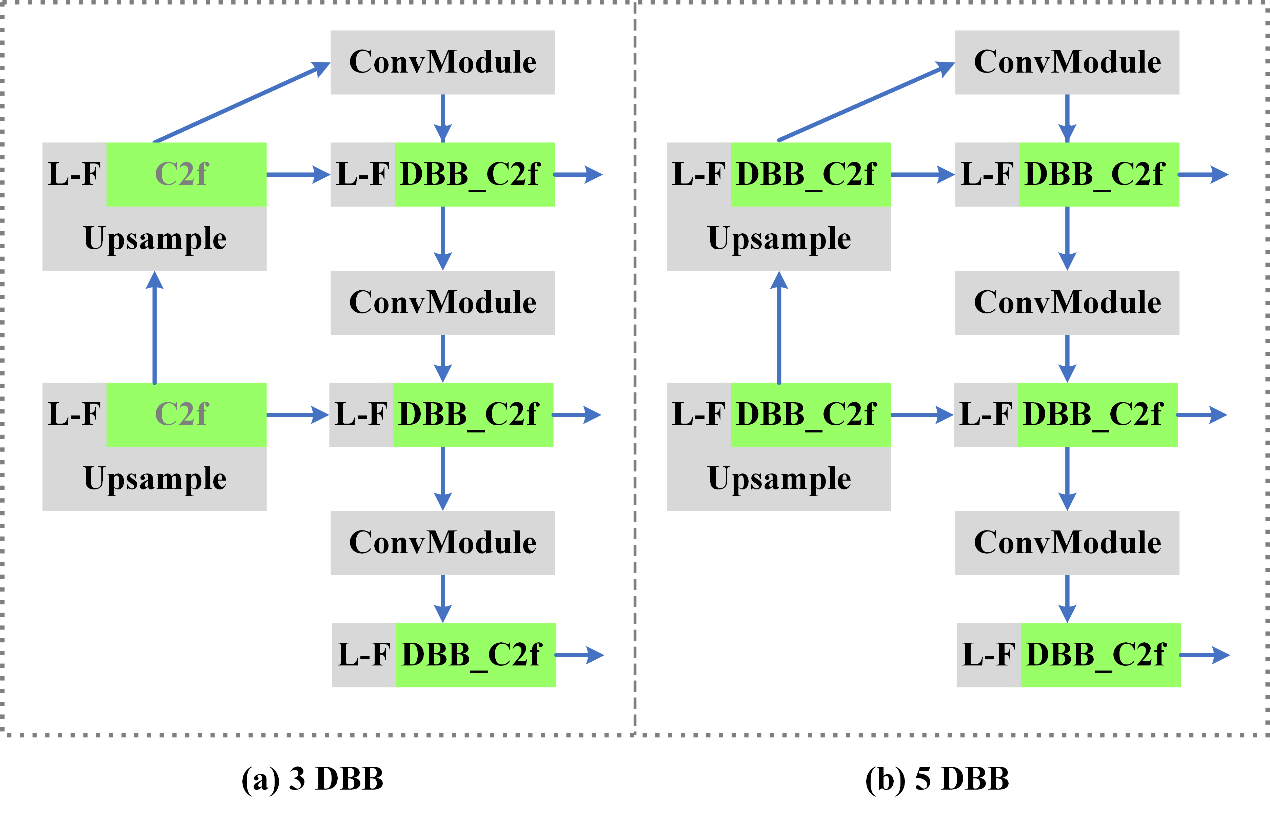
 **Supplementary Figure S4. Structure of Two BiFPN-Based Variants**

**Supplementary Table S5**

COMPARISON OF DIFFERENT FUSION APPROACHES

| No. | Fusion scheme | P | R | mAP_50_ | Parameters |
| --- | --- | --- | --- | --- | --- |
| 1 | Only C2f_DBB | 83.9 | 70.6 | 79.3 | 3.29M |
| 2 | Only BiFPN | 83.0 | 71.5 | 79.5 | 2.78M |
| 3 | BiFPN+3DBB | 80.4 | 71.7 | 79.2 | 3.05M |
| 4 | BiFPN+5DBB | 80.1 | 72.9 | 80.1 | 3.07M |

## Supplementary Section S5 Heatmap Analysis of Feature Synergy

To provide qualitative evidence for the synergistic effect between the Diverse Branch Block (DBB) and the Bidirectional Feature Pyramid Network (BiFPN), feature activation heatmaps were visualized for different model variants. The Grad-CAM technique was used for this visualization. The heatmaps were generated by averaging activations from the outputs of the three C2f modules that directly feed into the P3, P4, and P5 detection heads. The results are presented in Supplementary Figure S5. They visually corroborate the quantitative findings of the ablation study and help elucidate the mechanism behind the final model's enhanced performance.


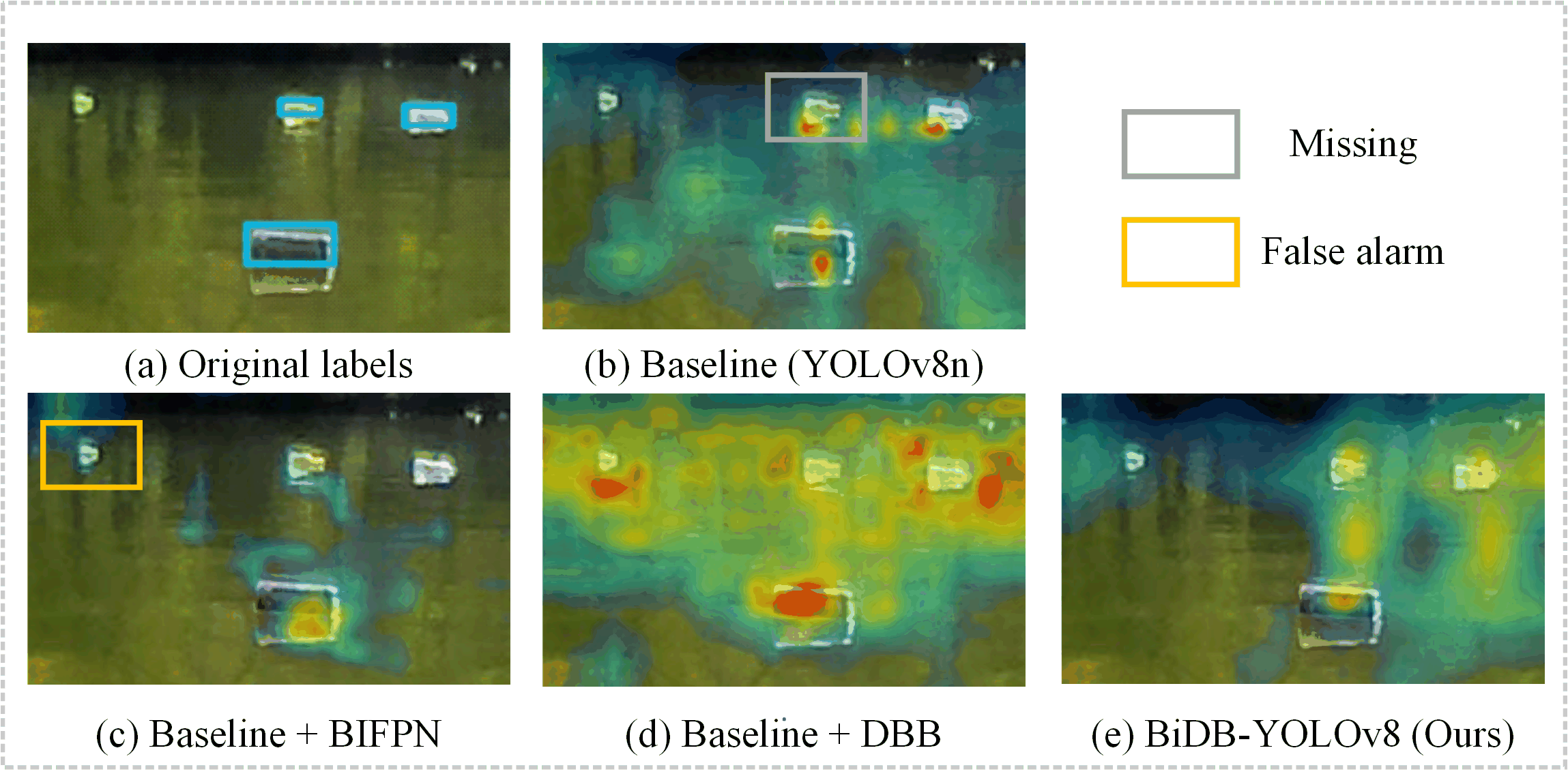


**Supplementary Figure S5. Visualization of feature activation heatmaps.** The heatmaps reveal how different architectural components influence the model's focus. Warmer colors (red) indicate higher network attention.

The analysis of the heatmaps reveals a clear progression in feature representation. The baseline model's activation (Panel b) is diffuse, with significant attention scattered across irrelevant background regions. This lack of focus corresponds to its missed detection ("Missing" box), confirming a limited feature extraction capability. The introduction of individual components addresses specific weaknesses. While BiFPN (Panel c) improves feature localization, it cannot fully compensate for the poor initial features, resulting in a "False Alarm." Conversely, the DBB module (Panel d) substantially enhances feature sensitivity, capturing all targets, but its activation is overly widespread, indicating that the richly extracted features are not efficiently fused.

The final proposed model, BiDB-YOLOv8 (Panel e), demonstrates a clear synergy. It inherits the heightened feature sensitivity of DBB while leveraging the precise localization capability of BiFPN. The resulting heatmap shows intense activation that is highly concentrated on the true targets, with background noise effectively suppressed. This visual evidence confirms that the synergistic combination of enhanced feature extraction and efficient fusion is key to the model's robustness, enabling it to achieve the best detection performance without errors.

Cheng, Y., Zhu, J., Jiang, M., Fu, J., Pang, C., Wang, P., Sankaran, K., Onabola, O., Liu, Y., Liu, D., 2021. Flow: A dataset and benchmark for floating waste detection in inland waters, Proceedings of the IEEE/CVF international conference on computer vision. pp. 10953-10962.

Yang, M., Qiao, G., Wang, H., Jiang, Y., 2023. Water Surface Floating Objects Dataset (IWHR_AI_Lable_Floater_V1) [DB/OL]. China Institute of Water Resources and Hydropower Research, <http://123.56.14.89:8008/wfdownload/>.
